# Updates to the Spectrum/AIM model for estimating key HIV indicators at national and subnational levels

**DOI:** 10.1097/QAD.0000000000002357

**Published:** 2019-09-05

**Authors:** John Stover, Robert Glaubius, Lynne Mofenson, Caitlin M. Dugdale, Mary-Ann Davies, Gabriela Patten, Constantin Yiannoutsos

**Affiliations:** a Avenir Health, Glastonbury, Connecticut; b Elizabeth Glaser Pediatric AIDS Foundation, Washington DC; c Medical Practice Evaluation Center and Division of Infectious Diseases, Massachusetts General Hospital, Boston, Massachusetts, USA; d Centre for Infectious Disease Epidemiology and Research, School of Public Health and Family Medicine, University of Cape Town, South Africa; e Indiana University Fairbanks School of Public Health, Indianapolis, Indiana, USA.

**Keywords:** estimates, HIV, model

## Abstract

**Background:**

The Spectrum/AIM model is used by national programs and UNAIDS to prepare annual estimates of the status of the HIV epidemic in 170 countries. The model and assumptions are updated regularly under the guidance of the UNAIDS Reference Group on Estimates, Modelling and Projections in response to new data, studies and program needs. This article describes the most recent updates for the 2018 round of estimates.

**Methods:**

New data on AIDS-related mortality from Europe and Brazil have been used to update mortality rates of those not on antiretroviral therapy (ART). Household survey data and new studies of pregnant women, mothers, and children have been used to improve estimates of the number of HIV-positive pregnant and breastfeeding women and pediatric ART initiation. New tools to estimate geographic variation in HIV prevalence have been used to prepare district estimates of key indicators.

**Results:**

The 2018 version of Spectrum includes: new estimates of non-ART AIDS-related mortality by CD4^+^ count that depend on ART coverage; a procedure to estimate country-specific patterns of HIV incidence by age by fitting to prevalence by age from household surveys; an updated estimate of postpartum transmission with ART started before pregnancy of 0.023% per month; an updated estimate of retention on treatment at delivery of 80% for all women on ART; a somewhat older pattern of ART initiation by age that has 26% of new pediatric patients initiating ART at 10–14 years of age, 18% at 2–4 years of age, and 26% at 5–9 years of age; and a new tool for estimating key HIV indicators at the district level.

**Conclusion:**

The new methods and data implemented in the 2018 version of Spectrum allow national programs more flexibility in describing their programs and are intended to improve the estimates of adult mortality and pediatric HIV indicators.

## Abstract 2

Supplemental Digital Content is available in the text

## Introduction

The Spectrum software (Avenir Health, Glastonbury, Connecticut, USA) is used to prepare estimates of key HIV indicators by national programs, UNAIDS and other international organizations. The model uses estimated incidence trends developed by fitting to HIV prevalence data [1,2] and then tracks the newly infected population by age, sex, CD4^+^ count category and antiretroviral therapy (ART) status. The model relies on country-specific service statistics, survey and surveillance data, and uses epidemiological parameters derived from scientific studies to inform patterns of incidence, progression, mortality, and fertility. Updates to some of these parameters are described in this article, others have been described previously. Table 1 provides a list of the key parameters used.

**Table 1:** Key parameters in Spectrum and sources for default values.

Epidemiological process	Source	Reference
Adult model		
Annual rate of decline in CD4^+^ counts	Fit to survival curves from ALPHA Network	[3]
Distribution of new infections by CD4^+^ count	Fit to survival curves from ALPHA Network	[3]
HIV mortality without ART		This article
Mortality on ART	IeDEA Consortium	[4]
Fertility of HIV-positive women	Household surveys	[5,6]
Incidence rate ratios by age	Household surveys	This article
Pediatric model		
Mother-to-child HIV transmission	Transmission studies	This article
Annual rate of decline in CD4^+^ percentage or count	Fit to ALPHA Network pediatric survival curves	[3,7]
Distribution of HIV-infected infants by CD4^+^ percentage	HIV Paediatric Prognostic Markers Collaborative Study [HPPMCS]	[8]
HIV mortality without ART	Fit to ALPHA Network pediatric survival curves	[3,7]
HIV mortality with ART	IeDEA Consortium	[9]
Age distribution of new ART patients	IeDEA Consortium	This article
Effect of cotrimoxazole on HIV-mortality	CHAP Trial, ARROW Trial	[10,11]

ART, antiretroviral therapy.

## AIDS mortality for people not receiving antiretroviral therapy

Spectrum tracks adults living with HIV by CD4^+^ count category (>500, 350–500, 250–349, 200–249, 100–199, 50–99, and <50 cells/μl). For those not on ART, the rates of mortality by CD4^+^ count and progression to lower CD4^+^ categories were determined by fitting a progression model to survival curves from the ALPHA Network cohort data from the pre-ART period [2,12,13] The estimated annual mortality rates are shown in Table 2. Note that these rates imply that even at high CD4^+^ counts, some people still die from HIV-related causes. Today, with a large portion of the HIV population on ART, the mortality rates for those not on treatment could be different if the chance of starting treatment is related to the likelihood of death (i.e. those most likely to die are also most likely to start treatment). Data from Western Europe and North America indicate that in 2017 there were 13 000 (9900–18 000) AIDS-related deaths out of an estimated population of people living with HIV (PLHIV) of 2.2 (1.9–2.4) million [14], an annual mortality rate of just 0.6%. ART coverage in these settings is high, at 78% (60%–90%), but about 20% of PLHIV are still without treatment. Even if all mortality occurred in those not on ART, the mortality rate would be only 2.7%/year. A likely explanation is that people who would have died without treatment, regardless of CD4^+^ count, are more likely to start treatment than those who are less likely to die. As a result, the people who are not on ART are the least likely to die. In that case, mortality rates from the pre-ART era will overestimate mortality rates today. We have accounted for this relationship in Spectrum by reducing the mortality rate for adults not on ART in each CD4^+^ category as the ART coverage rate in that CD4^+^ category increases.

**Table 2:** Annual rates of HIV-related mortality for people living with HIV and not on antiretroviral therapy by CD4^+^ category and age group.

	Age (years)			
CD4^+^ count (cells/μl)	15–24	25–34	35–44	45+
>500	0.005	0.004	0.005	0.005
350–500	0.011	0.010	0.013	0.013
250–349	0.026	0.026	0.036	0.032
200–249	0.061	0.069	0.096	0.080
100–199	0.139	0.185	0.258	0.203
50–99	0.321	0.499	0.691	0.513
<50	0.737	1.342	1.851	1.295

*μ′*_c_ = *μ*_c_ × (1 − ART coverage_c_), where *μ* is the annual pre-ART mortality rate of PLHIV not on ART in CD4^+^ category ‘c’ and *μ′* is the adjusted mortality rate.

The effect of this adjustment on mortality rates is small when ART coverage is low and large when ART coverage is high. The Kenya AIDS Indicator Survey of 2012 [15] reported ART coverage among all HIV-infected adults of about 35%, and that 31% of HIV-infected adults not on ART had CD4^+^ counts below 350 cells/μl, 15% had CD4^+^ counts 350–500 cells/μl, and 55% CD4^+^ counts above 500 cells/μl. With no adjustment, the overall annual mortality rate would be about 13% among those not on ART and about 2.3% for those on ART, yielding a combined annual mortality rate of 9%. With the mortality adjustment described here, the average annual mortality rate for those not on ART would drop to about 8% and the combined mortality rate to 6%. If ART coverage were 80% and those not on ART had the same CD4^+^ count distribution, then the annual mortality rate for those not on ART would drop to 2.5% and the overall annual mortality rate would be 2.3%. (Non-HIV-related mortality is accounted for separately in the model.)

France reported 90–120 AIDS deaths for 2014, 2015, and 2016 even though ART coverage was estimated at only about 80%. The model estimated over 600 AIDS deaths without the adjustment to non-ART mortality and about 200 with the change.

## Incidence by age

In 2016, 10% of Spectrum files estimated antiretroviral coverage for prevention of mother-to-child HIV transmission (PMTCT) at over 100%, which may indicate that Spectrum calculations underestimated PMTCT need. PMTCT need depends directly on HIV prevalence among reproductive-age women, and so on HIV incidence patterns by age. Spectrum uses incidence rate ratios (IRRs) to disaggregate adult (ages 15–49 or 15+) HIV incidence by age and sex [16]. Analysis of files with high PMTCT coverage revealed that Spectrum's default IRRs for generalized epidemics often underestimated HIV prevalence among young women relative to HIV prevalence in national surveys. For the 2017 HIV estimates, we developed a tool in Spectrum that adjusts IRRs to better fit HIV prevalence patterns in these surveys.

Spectrum utilizes age-specific and sex-specific IRR patterns. Sex-specific IRRs (sIRRs) quantify the female-to-male incidence ratio over time. Users may choose a default sIRR trend for generalized or concentrated epidemics or estimate it externally and input it manually [17]. The IRR fitting tool multiplies this input trend by a fitted scale factor *ϕ*.

We model the age-specific IRR (aIRR) at age *x*, relative to baseline age *b* = 27.5, as a shifted lognormal density [18],
 
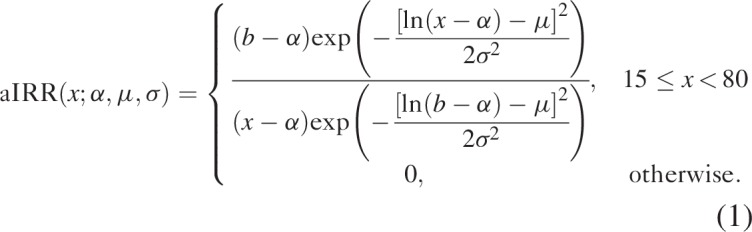


We fit aIRRs by tuning the parameters *α*, *μ*, and *σ*. We calculate aIRRs for each 5-year age group using the midpoint of each interval, for example, age 17.5 for the 15–19-year age group.

Fitted aIRRs may be either fixed or varying over time. Fixed aIRRs use the same age pattern of incidence over time. Time-varying aIRRs use one age pattern at the time of each survey; we linearly interpolate these patterns between surveys, and extrapolate by keeping the age pattern constant before the earliest survey and after the latest survey.

We fit IRRs within a Bayesian framework. We used the Nelder–Mead simplex method [19] to maximize the product of (a) a likelihood based on HIV prevalence survey data and (b) a prior distribution informed by incidence patterns from cohort studies. HIV prevalence survey data used for fitting consists of the number of respondents who tested HIV positive out of the total number tested by sex and 5-year age group. We derived the likelihood by assuming the number who tested positive was binomially distributed given the number tested and the model's HIV prevalence estimate in that age/sex group. We fitted IRR patterns to all surveys simultaneously. We accounted for complex survey design by dividing numbers of respondents by the survey design effect.

Bayesian parameter estimation requires placing prior probability distributions on model parameters based on external data or domain knowledge, so that parameter estimates incorporate this information alongside survey data. Our prior distribution only requires that *ϕ* is positive. We developed a data-driven prior probability distribution on aIRRs. We first used Incremental Mixture Importance Sampling [20] to sample aIRRs consistent with HIV incidence patterns at six ALPHA network sites in Malawi, Tanzania, South Africa, Uganda, and Zimbabwe [21]. The IRR fitting tool uses a mixture model of these site-specific incidence patterns as its prior distribution on incidence by age. We specify this prior as a mixture of multivariate lognormal distributions on *α*, *μ*, and *σ* for each sex, with one component of the mixture model corresponding to each ALPHA network site. This prior ensures that Spectrum produces realistic incidence patterns for settings similar to those observed at ALPHA network sites, whenever survey data are weak.

**Fig. 1:**
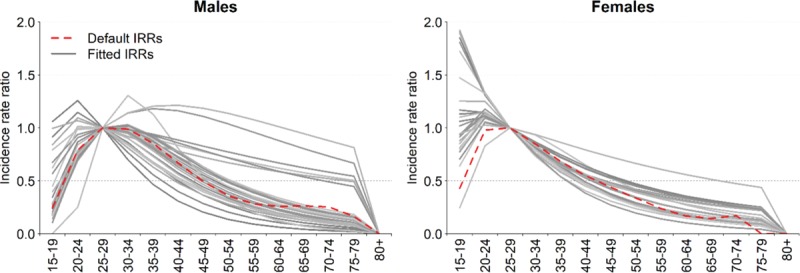
Fitted age-specific incidence rate ratios for 27 countries in sub-Saharan Africa.

We fitted IRRs separately for men and women for 27 countries with national HIV prevalence surveys. We fitted both fixed and time-varying IRRs in countries with multiple surveys, and selected the IRR model with lower Akaike information criterion (AIC). Figure 1 shows the resulting fitted aIRRs. Time-varying IRRs had lower AIC in four countries, whereas fixed IRRs had lower AIC in 23 countries. Whereas Spectrum's default generalized epidemic pattern suggests HIV incidence peaks at ages 25–34 for men and 25–29 for women, newly fitted incidence patterns usually peaked at younger ages. The better fits to the survey age pattern of prevalence improves the estimates of the number of women needing PMTCT services as fertility is concentrated in the younger ages.

Spectrum's default pattern for generalized epidemic settings is shown in red. Incidence rate ratios in year 2000 are shown for the four countries in which time-varying IRRs were used. IRRs, incidence rate ratios.

The resulting IRRs are applied on the incidence trends estimated by EPP (which can be for adults 15–49 or 15+) to distribute incidence by age and sex while leaving incidence 15–49 (or 15+) unchanged.

## Mother-to-child transmission rates

In 2015, a review of existing literature identified 48 new studies (in addition to 32 from the original 2012 review) to determine the probability of mother-to-child transmission for women receiving no antiretroviral (ARV) drugs and those receiving different ARV regimens to update transmission probabilities for the 2016 Spectrum model [9,22]. In 2018, an additional 24 publications and 3 abstracts were added to the 2016 analysis (see Annex for full listing of studies used for this analysis and a further explanation of regimen categories). The largest number of new studies were related to peripartum transmission rates from women starting ART during pregnancy (11 studies in formula-feeding, 7 in breastfeeding populations) and from women starting ART before pregnancy (10 studies in formula-feeding, 1 in breastfeeding populations). The updated transmission probabilities are included in the 2019 Spectrum model.

In the Spectrum model, transmission may occur *in utero* or intrapartum. These rates are combined to represent peripartum transmission. Peripartum also includes early postnatal transmission to age 4–6 weeks. Postnatal transmission is defined as transmission occurring in breastfeeding populations after age 4–6 weeks. The model calculates the numbers of new infections separately for peripartum and postnatal HIV transmission. Breastfeeding patterns are based on children receiving any type of breastfeeding (exclusive or nonexclusive) by age as reported in national household surveys.

**Fig. 2:**
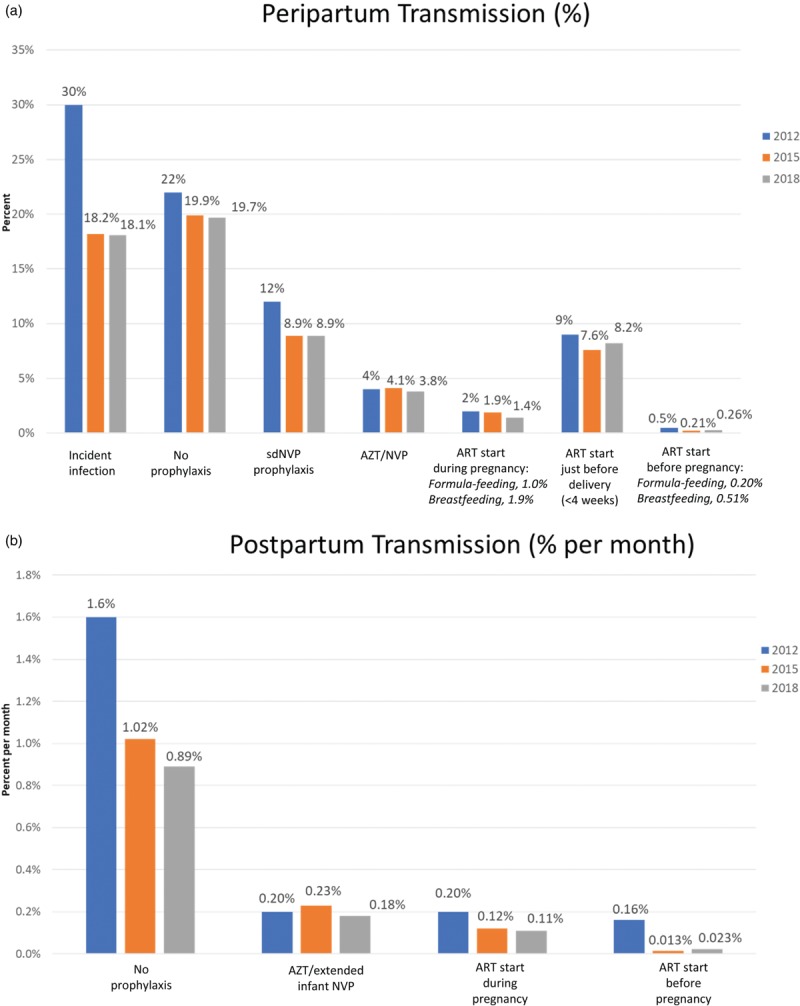
Probability of HIV transmission by antiretroviral regimen 2012, 2015, and 2018 review.

Figure 2 presents the changes to the probability of transmission between the 2012, 2015 and 2018 round of estimates. The peripartum values describe the probability of transmission during pregnancy and delivery (and early breastfeeding period in breastfeeding populations), and reflect levels of adherence existing in the study settings (for some studies, adherence may be better than adherence in the general population). The postpartum values are monthly probabilities that would be multiplied by the approximate duration of breastfeeding in months. Transmission because of an incident infection during breastfeeding is modeled as a one-time risk, and is thus not included in Fig. 2. The transmission probability for an incident infection during breastfeeding remains 27% (same as the 2015 model).

Peripartum transmission is the probability of transmission between a mother and child during the pregnancy and delivery (and if breastfeeding, including early breastfeeding to 4–6 weeks). Women starting ART during or before pregnancy who are breastfeeding have a slightly higher peripartum rate compared with those formula feeding. Postpartum transmission is a monthly probability (after age 4–6 weeks) of transmission that is multiplied by the expected duration of breastfeeding, which may vary for women within PMTCT programs and women not in PMTCT programs who are not likely to know their HIV status. The probability of transmission to the infant among women with incident infection while breastfeeding is a one-time risk and is 27% (the same as in the 2015 model). The sources for the 2018 model assumptions are Rollins *et al.* [17], Mahy *et al.* [4], and an unpublished report for the fall 2018 meeting of the UNAIDS Reference Group on Estimates, Modelling and Projections by Lynne Mofenson (see Annex). ART, antiretroviral therapy; AZT, zidovudine; NVP, nevirapine; PMTCT, prevention of mother-to-child transmission; sdNVP, single-dose nevirapine.

The review did not identify major changes from prior estimates, with differences between 2015 and 2018 in the decimal-point range. The greatest difference was for the estimate of postpartum transmission with ART started before pregnancy (0.013%/month in 2015 based on a small number of studies, with additional data increased to 0.023%/month in 2018). Additionally, estimated peripartum transmission rates in women on ART (started during pregnancy or preconception) were slightly higher in breastfeeding than formula-feeding populations, likely secondary to the contribution of early postpartum transmission to peripartum transmission in breastfeeding populations.

## Maternal retention in care

We reviewed the published literature (PubMed, EMBASE, and Web of Science, 2012–2018) and conference abstracts (CROI and International AIDS Society Meetings, 2016–2018) to inform estimates of retention in HIV care through pregnancy and the postpartum period among women on lifelong ART. Of women registering for antenatal HIV care, we derived the proportion retained in care at delivery. Of women in care at delivery, we derived the proportion retained in care at 6–10 weeks, and 6, 12, 18, and 24 months postpartum, irrespective of breastfeeding practices. From 24-month retention data, we derived a constant rate of postpartum loss to follow-up (LTFU) and translated this rate to a monthly risk.

We identified 39 unique references (supplementary appendix) describing 62 351 women from 13 low-income and middle-income countries. One study lacked data from any timepoints of interest [23]. Prior Spectrum values for maternal retention at delivery were stratified by timing of ART initiation (preconception: 75%; during pregnancy: 80%). In the updated literature review, we found limited evidence to support different retention for these two groups (supplementary appendix). The pooled proportion of women retained in care at delivery was 80%. Pooled postpartum retention among women in care at delivery was 67, 77, 74, 66, and 68% at 6–10 weeks, and 6, 12, 18, and 24 months, yielding a postpartum LTFU probability of 1.6% per month.

Definitions of retention in care varied substantially between studies, potentially influencing these pooled estimates [24]. Silent clinic transfers are frequent among pregnant and postpartum women [25,26]. Most studies did not report on clinic transfers, or classified transferred patients as nonretained, potentially underestimating retention in care. Consistent definitions of retention and more complete ascertainment of silent transfers and postpartum maternal mortality are needed to better refine estimates of retention in care throughout the PMTCT continuum.

## Age at antiretroviral therapy initiation among pediatric patients

As in previous Spectrum AIM model estimates, the age distribution of children initiating ART was adjusted to match the age distributions among children living with HIV (CLHIV) at ART start in the International Epidemiological Databases to Evaluate AIDS (IeDEA) Consortium (www.iedea.org). We included data from 85 924 ART-naïve children aged less than 15 years at ART initiation, with a recorded birth date and sex, who initiated ART between 2005 and 2016 in six IeDEA regions: Asia-Pacific (4% of CLHIV); Caribbean, Central and Latin America (1%); and West (5%), Southern (68%), Central (2%) and East (19%) Africa regions. Children who were transferred from other clinics were excluded because of uncertainty about the date of ART start. Overall, there was an increasing trend in the proportion of children initiating ART under age 2 years and over age 10 years. The proportion initiating ART under age 2 years increased from 24% in 2005–2007 to 29% in 2014–2016, consistent across regions except for West Africa where around a quarter of children were less than 2 years old at ART initiation throughout. Overall, the proportion of CLHIV initiating ART at age at least 10 years increased from 18% in 2005–2007 to 26% in 2014–2016, although in East Africa, this proportion remained constant at 26% and in Latin America it decreased from 25 to 17%. Correspondingly, the overall proportion initiating ART aged 2–4 years and 5–9 years decreased from 25 to 18% and 33 to 26% respectively, with consistent trends across all regions except for Latin America where the proportion aged 2–4 years increased from 2005–2007 to 2014–2016. Details are shown in Table 3.

**Table 3:** Number and percentage of children less than 15 years starting antiretroviral therapy in each age band by calendar period in the International Epidemiology Databases to Evaluate AIDS Global Collaboration.

	Number of children					Percentage of children per calendar period			
Calendar period	Age group in years					Age group in years			
All regions	Total	0–1	2–4	5–9	10–14	0–1	2–4	5–9	10–14
2005–2007	17 775	4188	4495	5883	3209	24%	25%	33%	18%
2008–2010	25 731	7436	5790	7583	4922	29%	23%	29%	19%
2011–2013	24 090	7051	4948	6760	5331	29%	21%	28%	22%
2014–2016	18 328	5376	3378	4842	4732	29%	18%	26%	26%
Africa – Central, East, Southern and West									
2005–2007	16 350	3904	4074	5365	3007	24%	25%	33%	18%
2008–2010	24 248	7024	5350	7151	4723	29%	22%	29%	19%
2011–2013	22 889	6730	4644	6375	5140	29%	20%	28%	22%
2014–2016	17 744	5211	3255	4658	4620	29%	18%	26%	26%
South and South-East Asia									
2005–2007	1198	226	373	454	145	19%	31%	38%	12%
2008–2010	1236	348	389	364	135	28%	31%	29%	11%
2011–2013	911	208	250	319	134	23%	27%	35%	15%
2014–2016	463	121	89	161	92	26%	19%	35%	20%
Caribbean, Central and South America									
2005–2007	227	58	48	64	57	26%	21%	28%	25%
2008–2010	247	64	51	68	64	26%	21%	28%	26%
2011–2013	290	113	54	66	57	39%	19%	23%	20%
2014–2016	121	44	34	23	20	36%	28%	19%	17%

Likely explanations for these patterns include: overall reduced numbers of new infant infections in recent calendar years because of increased coverage of effective PMTCT interventions; scale-up of early infant diagnosis and WHO guidelines recommending universal ART for all children aged less than 2 years in 2010 and 5 years in 2013; and backlog of undiagnosed HIV in long-term slow progressors aged at least 10 years who make up a relatively larger proportion of CLHIV in the context of fewer new infant infections, as well as adolescents with nonperinatally acquired HIV.

As previously described, the IeDEA data were transformed into age-specific probabilities of initiating ART among HIV-positive children not on ART [27].

## District estimates

Most countries apply the Spectrum model at the national level, but in 2018, 12 countries created separate Spectrum files for each province or state in the country (Benin, China, Côte d’Ivoire, Ethiopia, Haiti, India, Kenya, Mozambique, Nigeria, Togo, Zambia, and Zimbabwe). Detailed planning of interventions and resource allocation often happens at the next lowest level (districts). Although Spectrum can be applied at the district level, there are a number of challenges to doing so: demographic projections are more difficult because of the increased importance of migration, surveys may not be powered to provide reliable estimates at district level, and the number of individual Spectrum files required would be large and difficult to manage. Several approaches have been developed to estimate HIV prevalence at the district level, including those that apply geospatial techniques to survey data [28,29] and those that use small area estimation (SAE) techniques [30]. These approaches estimate HIV prevalence at the district level, but not other indicators of interest, such as AIDS deaths and new infections by age and sex.

In order to support district-level planning, we have added a District Estimation Tool to Spectrum. Currently, the tool provides district estimates for PLHIV, new infections, AIDS deaths, number on ART, ART coverage, and PMTCT coverage. The tool works by disaggregating the national or provincial estimates from the Spectrum files to the districts within the nation or province such that the sum of the districts always matches the Spectrum totals. The disaggregation by district of PLHIV, new infections, and AIDS deaths is done on the basis of the distribution of PLHIV between the ages of 15 and 49 years. The number on ART is disaggregated on the basis of the number of adults (age 15+ years) or children receiving ART. To produce these estimates, the tool requires inputs on the age distribution of the population aged 15–49 years, HIV prevalence among those aged 15–49 years, numbers of adults on ART, numbers of children on ART, and number of women receiving PMTCT services by district. The number of adults and children on ART and the number of women receiving PMTCT should be available from the Health Management Information System (HMIS). The size of the population aged 15–49 years by district can be based on the latest census. Prevalence by district may come from several sources including a geospatial model, such as HIVE or SAE techniques described in the previous paragraph, or from prevalence at antenatal clinics as reported in the HMIS.

The tool first calculates the proportion of people living with HIV (or taking adult ART or pediatric ART) aged 15–49 years at the provincial or national level that are in each district. These proportions are then used to disaggregate the other Spectrum indicators. The same distributions are applied to any age and sex group.

This tool provides detailed district-level estimates of key HIV indicators for HIV planning. It is designed to be useful for PEPFAR planning by providing key inputs into the develop of country operational plans. The main advantages of the tool are that it uses the official Spectrum estimates, ensures that all district estimates add up to the official provincial and national estimates, and provides a range of key indicators and sex/age groups. The main limitation is that it applies the same proportion distribution by district to each indicator. The distribution probably is different by indicator, but little information is available on which to base estimates.

## Conclusion

The updates to the Spectrum/AIM model use newly available data from surveys and epidemiological studies and are intended to improve the estimation of HIV epidemics. The updates described in this article, along with those described in other articles in this supplement, make better use of available research and survey information to improve the estimation of HIV-related mortality, incidence, and mother-to-child transmission and provide for national and subnational estimates. The most important effects are expected to be improved estimates of the coverage of PMTCT services (through more precise estimates of the number of HIV-infected pregnant women) and the burden of pediatric HIV and adult mortality.

## Acknowledgements

We acknowledge the patients, caregivers, sites and data centre staff of the International Epidemiology Databases to Evaluate AIDS (IeDEA) Collaboration. IeDEA is supported by the U.S. National Institutes of Health's National Institute of Allergy and Infectious Diseases, the Eunice Kennedy Shriver National Institute of Child Health and Human Development, the National Cancer Institute, the National Institute of Mental Health, and the National Institute on Drug Abuse: *Asia-Pacific*, U01AI069907; *CCASAnet*, U01AI069923; *Central Africa*, U01AI096299; *East Africa*, U01AI069911; *Southern Africa*, U01AI069924; *West Africa*, U01AI069919. This work is solely the responsibility of the authors and does not necessarily represent the official views of any of the institutions mentioned above.

### Conflicts of interest

There are no conflicts of interest.

## Supplementary Material

Supplemental Digital Content
