# Supplementary material for: Updates to the Spectrum/AIM model for estimating key HIV indicators at national and subnational levels
Source: AIDS. 2019 Sep 5;33(Suppl 3):S227–34. doi: 10.1097/QAD.0000000000002357 (PMC6919230; doi:10.1097/QAD.0000000000002357)
Supplement: Supplemental Digital Content [file aids-33-s227-s001.docx]

**Supplementary Material**

**Studies included in the review of mother to child transmission probabilities**

Review of data since the last 2015 review included published literature, reviewed by Pub Med search, personal files, and conference presentations (CROI and IAS 2016-2018). Cohort and observational data as well as clinical trials were included. Criteria for inclusion included: 1) provision of data on timing of diagnostic testing to be able to distinguish peripartum and postpartum transmission timing; 2) delineation of type of maternal antepartum antiretroviral (ARV) regimen; 3) if mother received antiretroviral therapy (ART), timing of initiation (before/during pregnancy); 4) when available, data on HIV RNA levels and transmission if the mother was receiving ART during pregnancy and timing of RNA measurement was near delivery; 5) when available, data on duration of maternal ART and transmission.

The search terms included: “incident/acute HIV in pregnancy” (37 papers identified), “incident/acute HIV during breastfeeding” (5 papers identified), and “mother to child HIV transmission” (949 papers identified); 991 papers and 7 abstracts were identified, 86 of which underwent full review, yielding 24 relevant new publications and 3 abstracts (1-27).

*Peripartum HIV transmission* was measured by infant HIV status generally at 4-6 weeks of age, although some studies reported data at 2 weeks or as late as 3 months. In formula-fed infants, this reflects in utero and intrapartum transmission; in breast-fed infants this reflects in utero, intrapartum and early postpartum transmission. The cumulative number of infections was divided by the cumulative number of HIV-exposed infants for an average peripartum transmission rate for each ARV category.

*Postpartum HIV transmission* was measured by transmission rates in breastfed infants who were uninfected at 4-6 weeks and subsequently found to be infected. The age at which postpartum transmission was measured differed between studies; in the 2015 review, the data primarily reflected 6-month data, because most studies were reporting on interventions that ceased at 6 months. However, since guidelines now recommend ART for all pregnant and breastfeeding women, ART no lower stops at 6 months, and the more recent studies report on postnatal transmission for longer durations on ART, through 12-18 months. Monthly postnatal transmission probabilities were calculated by dividing the cumulative postnatal transmission percent measured over a particular time interval by the number of months in that time period after subtracting the number of months when the “baseline” transmission was measured (because early postpartum transmission would have already been included in the “peripartum” 4-6 week transmission rate). For example, if peripartum transmission was measured at 6 weeks and cumulative transmission was measured again at 6 months, the transmission rate at 6 months was subtracted from the 6-week transmission rate, and divided by 4.5 months (the period during which breastfeeding had occurred in the infant after 6 weeks). Thus, if transmission at 6 weeks was 4% and at 6 months was 8%, then 4% of transmission was attributed to breastfeeding and was divided by 4.5 months (the time period between the 6-week and 6-month measurement), giving a postnatal transmission rate of 0.89% per month of breastfeeding. The weighted average for the particular PMTCT regimen category was calculated based on the study sample size.

*Incident infection* refers to newly acquired infections in pregnant or lactating women. Methods used to identify such women varied between studies, in some cases reflecting seroconversion from HIV-negative to positive and in a few studies reflecting the use of specific assay values.

Studies to evaluate postpartum transmission for the “*no prophylaxis*” category included studies of infants whose mothers received no ARV during pregnancy and postnatally or had received very short course (<4 weeks) zidovudine (AZT) or AZT/lamivudine (3TC) antepartum regimens. Studies to evaluate postpartum transmission for the “*single-dose nevirapine (sdNVP) prophylaxis*” category included studies in which infants had received sdNVP prophylaxis and no further infant or maternal prophylaxis was given.

Studies to evaluate postpartum transmission with *AZT/NVP* included studies in which extended infant prophylaxis was given; this included when the mother had received antepartum AZT/intrapartum sdNVP or in a few cases when the mother had not received AZT/sdNVP (e.g., the Breastfeeding and Nutrition (BAN) study was postnatal infant prophylaxis alone) (28).

*Duration of antepartum ART* was defined by the study; only studies that included mothers starting ART during pregnancy were included for evaluation of duration of ART and transmission. The category of < or >4 weeks was analyzed (included as “ART start near delivery” for the Spectrum model), but only a four studies (one new) reported this timeline.

An analysis of peripartum transmission by *maternal viral load near delivery* in women receiving ART was also performed, with a focus on “high viral load” >1,000 copies/mL criteria, although some studies reported on different viral load thresholds (>50, >400 copies/mL). Of 11 studies allowing comparison of peripartum transmission with viral load >1,000 copies/mL, 8 were from resource-rich, formula-feeding countries. These estimates were not used in the revised Spectrum model due to limited data availability from low-middle income or breastfeeding settings to inform the model, and viral load data were not viewed as being readily available in low-middle income countries.

**References**

1. Flynn PM, Taha TE, Cababasay M, et al; PROMISE Study Team. [Prevention of HIV-1 transmission through breastfeeding: efficacy and safety of maternal antiretroviral therapy versus infant nevirapine prophylaxis for duration of breastfeeding in HIV-1-infected women with high CD4 cell count (IMPAACT PROMISE): A randomized, open-label, clinical trial.](https://www.ncbi.nlm.nih.gov/pubmed/29239901) *J Acquir Immune Defic Syndr*. 2018;77:383-392.
2. Carey L, Desouza C, Moorcroft A, Elgalib A. [Pregnancy outcomes of women with HIV in a district general hospital in the UK.](https://www.ncbi.nlm.nih.gov/pubmed/29526132) *J Obstet Gynaecol*. 2018;38:1-4.
3. Salazar-Austin N, Hoffmann J, Cohn S, et al; TSHEPISO Study Team. [Poor obstetric and infant outcomes in human immunodeficiency virus-infected pregnant women with tuberculosis in South Africa: The Tshepiso Study.](https://www.ncbi.nlm.nih.gov/pubmed/29028970) *Clin Infect Dis*. 2018;66:921-929.
4. Dinh TH, Mushavi A, Shiraishi RW, et al. [Impact of timing of antiretroviral treatment and birth weight on mother-to-child human immunodeficiency virus transmission: findings from an 18-month prospective cohort of a nationally representative sample of mother-infant pairs during the transition from Option A to Option B+ in Zimbabwe.](https://www.ncbi.nlm.nih.gov/pubmed/29401270) *Clin Infect Dis*. 2018;66:576-585.
5. DolPHIN-1: Randomized controlled trial of dolutegravir versus efavirenz-based therapy in mothers initiating antiretroviral treatment in late pregnancy. International AIDS Conference, Amsterdam, the Netherlands, July 2018, Abstract THAB0307LB
6. Bornhede R, Soeria-Atmadja S, Westling K, Pettersson K, Navér L. [Dolutegravir in pregnancy-effects on HIV-positive women and their infants.](https://www.ncbi.nlm.nih.gov/pubmed/29396773) *Eur J Clin Microbiol Infect Dis*. 2018;37:495-500.
7. Grayhack C, Sheth A, Kirby O, et al. [Evaluating outcomes of mother-infant pairs using dolutegravir for HIV treatment during pregnancy.](https://www.ncbi.nlm.nih.gov/pubmed/29944472) *AIDS*. 2018;32:2017-2021.
8. Lamorde M, Wang X, Neary M, et al. Pharmacokinetics, pharmacodynamics and pharmacogenetics of efavirenz 400 mg once daily during pregnancy and postpartum. *Clin Infect Dis*. 2017;67:785-90.
9. Gill MM, Hoffman HJ, Ndatimana D, et al. [24-month HIV-free survival among infants born to HIV-positive women enrolled in Option B+ program in Kigali, Rwanda: The Kabeho Study.](https://www.ncbi.nlm.nih.gov/pubmed/29390577) *Medicine*. 2017;96:e9445.
10. Ørbaek M, Thorsteinsson K, Helleberg M, et al. [Assessment of mode of delivery and predictors of emergency caesarean section among women living with HIV in a matched-pair setting with women from the general population in Denmark, 2002-2014.](https://www.ncbi.nlm.nih.gov/pubmed/28544321) *HIV Med*. 2017;18:736-747.
11. Brites C, Nobrega I, Travassos AG, et al. Raltegravir vs lopinavir for late-presenters pregnant women. International AIDS Conference, Paris, France, July 2017, Abstract WEAC0201.

# [Schalkwijk S](https://www.ncbi.nlm.nih.gov/pubmed/?term=Schalkwijk%20S%5BAuthor%5D&cauthor=true&cauthor_uid=28595298), [Colbers A](https://www.ncbi.nlm.nih.gov/pubmed/?term=Colbers%20A%5BAuthor%5D&cauthor=true&cauthor_uid=28595298), [Konopnicki D](https://www.ncbi.nlm.nih.gov/pubmed/?term=Konopnicki%20D%5BAuthor%5D&cauthor=true&cauthor_uid=28595298), et al; [Pharmacokinetics of newly developed antiretroviral agents in HIV-infected pregnant women (PANNA) Network](https://www.ncbi.nlm.nih.gov/pubmed/?term=Pharmacokinetics%20of%20newly%20developed%20antiretroviral%20agents%20in%20HIV-infected%20pregnant%20women%20(PANNA)%20Network%5BCorporate%20Author%5D). Lowered rilpivirine exposure during the third trimester of pregnancy in human immunodeficiency virus type 1-infected women. [*Clin Infect Dis*.](https://www.ncbi.nlm.nih.gov/pubmed/28595298) 2017;65:1335-1341.

1. Myer L, Phillips TK, McIntyre JA, et al. [HIV viraemia and mother-to-child transmission risk after antiretroviral therapy initiation in pregnancy in Cape Town, South Africa.](https://www.ncbi.nlm.nih.gov/pubmed/27353189) *HIV Med*. 2017;18:80-88.
2. Scott GB, Brogly SB, Muenz D, Stek AM, Read JS; International Maternal Pediatric Adolescent AIDS Clinical Trials Group (IMPAACT) P1025 Study Team. [Missed opportunities for prevention of mother-to-child transmission of human immunodeficiency virus.](https://www.ncbi.nlm.nih.gov/pubmed/28277349) *Obstet Gynecol*. 2017;129:621-628.

# Peters H, Francis K, Sconza R, et al. UK mother-to-child HIV transmission rates continue to decline: 2012-2014. *Clin Infect Dis.* 2017;64:527-8.

# Tiam A, Kassaye S, Machekano R, et al. Shifting dynamics of HIV transmission timing among infants in the era of Option B+ and implications for infant testing. International AIDS Conference, July 2017, Paris, France, Abstract TUPEB0423.

1. Mulligan N, Schalkwijk S, Best BM, et al. [Etravirine pharmacokinetics in HIV-infected pregnant women.](https://www.ncbi.nlm.nih.gov/pubmed/27540363) *Front Pharmacol*. 2016;7:239.
2. Perry MEO, Taylor GP, Sabin CA, et al. Lopinavir and atazanavir in pregnancy: comparable infant outcomes, virological efficacies, and preterm delivery rates. *HIV Med*. 2016;17:28-35.
3. Goga AE, Dinh TH, Jackson DJ, et al; South Africa PMTCT Evaluation (SAPMCTE) Team. [Population-level effectiveness of PMTCT Option A on early mother-to-child (MTCT) transmission of HIV in South Africa: implications for eliminating MTCT.](https://www.ncbi.nlm.nih.gov/pubmed/27698999) *J Glob Health*. 2016;6:020405.

# [Olana T](https://www.ncbi.nlm.nih.gov/pubmed/?term=Olana%20T%5BAuthor%5D&cauthor=true&cauthor_uid=27617023), [Bacha T](https://www.ncbi.nlm.nih.gov/pubmed/?term=Bacha%20T%5BAuthor%5D&cauthor=true&cauthor_uid=27617023), [Worku W](https://www.ncbi.nlm.nih.gov/pubmed/?term=Worku%20W%5BAuthor%5D&cauthor=true&cauthor_uid=27617023), [Tadesse BT](https://www.ncbi.nlm.nih.gov/pubmed/?term=Tadesse%20BT%5BAuthor%5D&cauthor=true&cauthor_uid=27617023). Early infant diagnosis of HIV infection using DNA-PCR at a referral center: an 8 years retrospective analysis. [*AIDS Res Ther*.](https://www.ncbi.nlm.nih.gov/pubmed/?term=Olana+T) 2016;13:29.

# Tookey PA, Thorne C, van Wyk, Norton M. Maternal and foetal outcomes among 4118 women with HIV infection treated with lopinavir/ritonavir during pregnancy: analysis of population-based surveillance data from the national study of HIV in pregnancy and childhood in the United Kingdom and Ireland. *BMC Infect Dis.* 2016;16:65.

# Blonk MI, Colbers AP, Hidalgo-Tenorio C, et al; Pharmacokinetics of Newly Developed Antiretroviral Agents in HIV-Infected Pregnant Women PANNA Network; PANNA Network. [Raltegravir in HIV-1-Infected Pregnant Women: Pharmacokinetics, Safety, and Efficacy.](https://www.ncbi.nlm.nih.gov/pubmed/25944344) *Clin Infect Dis*. 2015;61:809-16.

1. Cohan D, Natureeba P, Koss CA, et al. [Efficacy and safety of lopinavir/ritonavir versus efavirenz-based antiretroviral therapy in HIV-infected pregnant Ugandan women.](https://www.ncbi.nlm.nih.gov/pubmed/25426808) *AIDS*. 2015;29:183-91.
2. Montgomery-Taylor S, Hemelaar J. [Management and outcomes of pregnancies among women with HIV in Oxford, UK, in 2008-2012.](https://www.ncbi.nlm.nih.gov/pubmed/25912413) *Int J Gynaecol Obstet*. 2015;130:59-63.
3. Colbers A, Best B, Schalkwijk S, et al; PANNA Network and the IMPAACT 1026 Study Team. [Maraviroc pharmacokinetics in HIV-1-infected pregnant women.](https://www.ncbi.nlm.nih.gov/pubmed/26202768) *Clin Infect Dis*. 2015;61:1582-9.
4. Colbers A, Moltó J, Ivanovic J, et al; PANNA Network. [Pharmacokinetics of total and unbound darunavir in HIV-1-infected pregnant women.](https://www.ncbi.nlm.nih.gov/pubmed/25326090) *J Antimicrob Chemother.* 2015;70:534-42.
5. Lima YA, Cardoso LP, Reis MN, Stefani MM. I[ncident and long-term HIV-1 infection among pregnant women in Brazil: Transmitted drug resistance and mother-to-child transmission.](https://www.ncbi.nlm.nih.gov/pubmed/27037910) *J Med Virol*. 2016;88:1936-43.

**Appendix Table 1. Retention in care at delivery.**

| **Study** | **Setting** | **# At risk** | **# Retained in care** | **% In care at delivery** | **% On ART**  **pre-conception^a^** |
| --- | --- | --- | --- | --- | --- |
| **Studies directly reporting on retention near delivery:** | | | | | |
| Abrams (2018) | Swaziland | 983 | 692 | 70% | 0% |
| Deschamps (2018) | Haiti | 883 | 657 | 74% | 42% |
| Oyeledun (2017) | Nigeria | 247 | 208 | 84% | 0% |
| Schnack (2016) | Uganda | 124 | 79 | 64% | 0% |
| **Studies from which retention near delivery was inferred^b^:** | | | | | |
| Chan (2016) | Malawi | 456 | 381 | 84% | 11% |
| Erlwanger (2017) | Malawi | 997 | 902 | 90% | 0% |
| Hauser (2018) | Malawi | 478 | 417 | 87% | 0% |
| Kim (2015) | Malawi | 1302 | 998 | 77% | 30% |
| Tweya (2014) | Malawi | 2930 | 2491 | 85% | 0% |
| **Pooled estimate:** | | | | | |
| **Total** |  | **8400** | **6825** | **81%** |  |
| **ANC:** antenatal care  **^a^** The pooled estimate of retention in care at delivery was 77% for the 3 studies that included any ART use prior to pregnancy and 83% for the six studies that did not include any ART use prior to pregnancy.  **^b^** These studies did not directly report retention at delivery; however, these studies did report retention in care at month X among women who were registered in antenatal care. If median gestational age at antenatal care enrollment was specified for an ART-naïve cohort, this information was used to approximate time of ART start. If median time on ART and/or gestational age at enrollment were not specified, it was assumed that women were registered in antenatal HIV care three months prior to delivery based on the average time on ART in pregnancy reported from the included studies. If the proportion retained in care was reported at month X and month X aligned with expected time of delivery based on gestational age at ANC enrollment and/or time on ART prior to delivery, then this proportion was taken to represent the % in care at delivery. | | | | | |

**Appendix Table 2. Retention in care through 24 months postpartum.**

| **2a. Retention in care at 6-10 weeks postpartum** | | | | |
| --- | --- | --- | --- | --- |
| **Study** | **Setting** | **# At risk^a^** | **# In care** | **% In care** |
| Asbjornsdottir (2017) | Mozambique | 1,576 | 851 | 54% |
| Dzangare (2016) | Zimbabwe | 118 | 96 | 81% |
| Etoori (2018) | Swaziland | 496 | 396 | 80% |
| Hauser (2018) | Malawi | 417 | 360 | 75% |
| Joseph (2016) | Uganda | 686 | 499 | 73% |
| Odeny (2018) | Kenya | 747 | 571 | 76% |
| Sarko (2017)^b^ | Nigeria | 168 | 14 | 8% |
| Schwartz (2015) | South Africa | 50 | 48 | 96% |
|  |  |  |  |  |
| **Pooled estimate** |  | **4,258** | **2,835** | **67%** |
| **2b. Retention in care at ~6m postpartum^c^** | | | | |
| **Study** | **Setting** | **# At risk^a^** | **# In care** | **% In care** |
| Abrams (2018) | Swaziland | 692 | 558 | 81% |
| Asbjornsdottir (2017) | Mozambique | 1,576 | 504 | 32% |
| Chan (2016) | Malawi | 381 | 368 | 85% |
| Erlwanger (2017) | Zimbabwe | 902 | 849 | 85% |
| Etoori (2018) | Swaziland | 496 | 351 | 71% |
| Ford (2017) | Zimbabwe | 385 | 327 | 85% |
| Foster (2017) | Zimbabwe | 138 | 113 | 82% |
| Harrington (2018) | Malawi | 291 | 235 | 81% |
| Koole (2014) | Malawi | 586 | 498 | 85% |
| Muhumuza (2017) | Uganda | 2,169 | 1,609 | 74% |
| Musomba (2017) | Uganda | 856 | 830 | 97% |
| Nance (2017) | Tanzania | 374 | 220 | 59% |
| Olwedo (2016) | Uganda | 277 | 177 | 64% |
| Oyeledun (2017) | Nigeria | 208 | 146 | 69% |
| Sarko (2017)^d^ | Nigeria | 149 | 10 | 7% |
| Tweya (2014) | Malawi | 2,491 | 2,403 | 82% |
|  |  |  |  |  |
| **Pooled estimate** |  | **12,853** | **9,198** | **77%** |

**Appendix Table 2. Retention in care through 24 months postpartum (cont).**

| **2c. Retention in care at ~12m postpartum^e^** | | | | |
| --- | --- | --- | --- | --- |
| **Study** | **Setting** | **# At risk^a^** | **# In care** | **% In care** |
| Akama (2018) | Kenya | 156 | 123 | 79% |
| Atanga (2017) | Cameroon | 211 | 182 | 86% |
| Deschamps (2018) | Haiti | 657 | 474 | 54% |
| Domercant (2017) | Haiti | 3,390 | 1,681 | 50% |
| Erlwanger (2017) | Zimbabwe | 902 | 753 | 76% |
| Ford (2017) | Zimbabwe | 382 | 302 | 79% |
| Gamell (2017) | Tanzania | 109 | 92 | 66% |
| Haas (2016) | Malawi | 26,658 | 20,475 | 77% |
| Kamuyango (2014) | Malawi | 189 | 185 | 98% |
| Karajeanes (2017) | Mozambique | 8,316 | 5,946 | 72% |
| Llenas-Garcia (2016) | Mozambique | 303 | 124 | 41% |
| CDC MMWR (2013) | Malawi | 2,949 | 2,267 | 77% |
| Muhumuza (2017) | Uganda | 2,169 | 1,447 | 67% |
| Mwapasa (2017) | Malawi | 384 | 274 | 71% |
| Myer (2018) | South Africa | 192 | 136 | 71% |
| Olwedo (2016) | Uganda | 277 | 114 | 41% |
| Phiri (2017) | Malawi | 437 | 261 | 60% |
| Schwartz (2015) | South Africa | 45 | 33 | 73% |
| Tweya (2014) | Malawi | 2,491 | 2,315 | 79% |
|  |  |  |  |  |
| **Pooled estimate** |  | **50,217** | **37,184** | **74%** |
| **2d. Retention in care at ~18m postpartum^f^** | | | | |
| **Study** | **Setting** | **# At risk^a^** | **# In care** | **% In care** |
| Bobrow (2016) | Rwanda | 575 | 458 | 80% |
| Etoori (2018) | Swaziland | 455 | 262 | 58% |
| Mikitu (2016) | Ethiopia | 346 | 268 | 78% |
| Muhumuza (2017) | Uganda | 2,169 | 1,345 | 62% |
|  |  |  |  |  |
| **Pooled estimate** |  | **3,545** | **2,333** | **66%** |
| **2e. Retention in care at ~24m postpartum^g^** | | | | |
| **Study** | **Setting** | **# At risk^a^** | **# In care** | **% In care** |
| Haas (2016) | Malawi | 25,849 | 18,306 | 71% |
| Karajeanes (2017) | Mozambique | 8,316 | 4,915 | 59% |
| Phiri (2017) | Malawi | 432 | 169 | 39% |
|  |  |  |  |  |
| **Pooled estimate** |  | **34,597** | **23,390** | **68%** |
| **^a^** When reported, deaths and clinic transfers were censored from the number at risk at each time point.  **^b^** The pooled estimate with removal of the Sarko (2017) study as an outlier is 69%.  **^c^** Pooled retention in care at ~6 months included data reported between the window of 3-8 months postpartum.  **^d^** The pooled estimate with removal of the Sarko (2017) study as an outlier is unchanged at 78%  **^e^** Pooled retention in care at ~12 months included data reported between the window of 9-14 months postpartum.  **^f^** Pooled retention in care at ~18 months included data reported between the window of 15-20 months postpartum.  **^g^** Pooled retention in care at ~24 months included data reported between the window of 21-16 months postpartum. | | | | |

**References included in the review of maternal retention at delivery and postpartum:**^1-39^

1. Abrams EJ, Langwenya N, Gachuhi A, et al. Impact of universal antiretroviral therapy for pregnant and postpartum women on antiretroviral therapy uptake and retention. *AIDS.* Jan 27 2019;33(1):45-54.

2. Akama E, Nimz A, Blat C, et al. Retention and viral suppression of newly diagnosed and known HIV positive pregnant women on Option B+ in Western Kenya. *AIDS Care.* Sep 27 2018:1-7.

3. Asbjornsdottir K, Rustagi A, Coutinho J, et al. Low retention in care among recently diagnosed women enrolled in Option B+ care in Mozambique. International AIDS Society Meeting; 2017; Paris, France.

4. Atanga PN, Ndetan HT, Achidi EA, Meriki HD, Hoelscher M, Kroidl A. Retention in care and reasons for discontinuation of lifelong antiretroviral therapy in a cohort of Cameroonian pregnant and breastfeeding HIV-positive women initiating "Option B+" in the South West Region. *Tropical Medicine & International Health.* 2017;22(2):161-170.

5. Centers for Disease Control. Impact of an innovative approach to prevent mother-to-child transmission of HIV--Malawi, July 2011-September 2012. *MMWR. Morbidity and Mortality Weekly Report.* 2013;62(8):148-151.

6. Chan AK, Kanike E, Bedell R, et al. Same day HIV diagnosis and antiretroviral therapy initiation affects retention in Option B+ prevention of mother-to-child transmission services at antenatal care in Zomba District, Malawi. *Journal of the International Aids Society.* 2016;19:6.

7. Deschamps MM, Jannat-Khah D, Rouzier V, et al. Fifteen years of HIV and syphilis outcomes among a prevention of mother-to-child transmission program in Haiti: from monotherapy to Option B+. *Tropical Medicine and International Health.* 2018;23(7):724-737.

8. Domercant JW, Puttkammer N, Young P, et al. Attrition from antiretroviral treatment services among pregnant and non-pregnant patients following adoption of Option B+ in Haiti. *Glob Health Action.* 2017;10(1):1330915.

9. Dzangare J, Takarinda KC, Harries AD, et al. HIV testing uptake and retention in care of HIV-infected pregnant and breastfeeding women initiated on "Option B+" in rural Zimbabwe. *Tropical Medicine & International Health.* 2016;21(2):202-209.

10. Erlwanger AS, Joseph J, Gotora T, et al. Patterns of HIV care clinic attendance and adherence to antiretroviral therapy among pregnant and breastfeeding women living with HIV in the context of option B+ in Zimbabwe. *Journal of Acquired Immune Deficiency Syndromes (1999).* 2017;75 Suppl 2:S198-S206.

11. Etoori D, Kerschberger B, Staderini N, et al. Challenges and successes in the implementation of option B+ to prevent mother-to-child transmission of HIV in southern Swaziland. *BMC Public Health.* 2018;18(1):374.

12. Ford D, Muzambi M, Nkhata MJ, et al. Implementation of antiretroviral therapy for life in pregnant/breastfeeding HIV+ women (option B+) alongside rollout and changing guidelines for ART initiation in rural Zimbabwe: The Lablite Project experience. *Journal of Acquired Immune Deficiency Syndromes (1999).* 2017;74(5):508-516.

13. Foster G, Orne-Gliemann J, Font H, et al. Impact of facility-based mother support groups on retention in care and PMTCT outcomes in rural Zimbabwe: The EPAZ cluster-randomized controlled trial. *Journal of Acquired Immune Deficiency Syndromes.* 2017;75:S207-S215.

14. Gamell A, Luwanda LB, Kalinjuma AV, et al. Prevention of mother-to-child transmission of HIV Option B plus cascade in rural Tanzania: The One Stop Clinic model. *PLoS One.* 2017;12(7):15.

15. Haas AD, Tenthani L, Msukwa MT, et al. Retention in care during the first 3 years of antiretroviral therapy for women in Malawi's option B plus programme: an observational cohort study. *Lancet HIV.* 2016;3(4):E175-E182.

16. Harrington BJ, Pence BW, Maliwichi M, et al. Probable antenatal depression at antiretroviral initiation and postpartum viral suppression and engagement in Option B. *AIDS.* Sep 17 2018.

17. Hauser BM, Miller WC, Tweya H, et al. Assessing Option B+ retention and infant follow-up in Lilongwe, Malawi. *International Journal of STD & AIDS.* 2018;29(2):185-194.

18. Joseph J, Suggu K, Hariharan N, et al. Increasing retention of HIV-positive pregnant and postnatal women and HIV-exposed infants: Measuring the effects of follow-up activities and improved patient management in rural Uganda. *Journal of the International AIDS Society.* 2016;19:25-26.

19. Kamuyango AA, Hirschhorn LR, Wang W, Jansen P, Hoffman RM. One-year outcomes of women started on antiretroviral therapy during pregnancy before and after the implementation of Option B+ in Malawi: A retrospective chart review. *World Journal of AIDS.* 2014;4(3):332-337.

20. Karajeanes E, Bila D, Augusto O, et al. Uptake and retention in care of pregnant women starting option B+ in Maputo. *Topics in Antiviral Medicine.* 2017;25(1):326s.

21. Kim MH, Ahmed S, Hosseinipour MC, et al. The impact of option B+ on the antenatal PMTCT cascade in Lilongwe, Malawi. *Journal of Acquired Immune Deficiency Syndromes.* 2015;68(5):E77-E83.

22. Koole O, Houben RM, Mzembe T, et al. Improved retention of patients starting antiretroviral treatment in Karonga District, northern Malawi, 2005-2012. *Journal of Acquired Immune Deficiency Syndromes (1999).* 2014;67(1):e27-e33.

23. Koss CA, Natureeba P, Kwarisiima D, et al. Viral suppression and retention in care up to 5 years after initiation of lifelong ART during pregnancy (option B+) in rural Uganda. *Journal of Acquired Immune Deficiency Syndromes.* 2017;74(3):279-284.

24. Llenas-Garcia J, Wikman-Jorgensen P, Hobbins M, et al. Retention in care of HIV-infected pregnant and lactating women starting ART under Option B+ in rural Mozambique. *Tropical Medicine & International Health.* 2016;21(8):1003-1012.

25. Mitiku I, Arefayne M, Mesfin Y, Gizaw M. Factors associated with loss to follow-up among women in Option B+ PMTCT programme in northeast Ethiopia: a retrospective cohort study. *Journal of the International AIDS Society.* 2016;19(1):20662.

26. Muhumuza S, Akello E, Kyomugisha-Nuwagaba C, et al. Retention in care among HIV-infected pregnant and breastfeeding women on lifelong antiretroviral therapy in Uganda: A retrospective cohort study. *PLoS One.* 2017;12(12):e0187605.

27. Musomba R, Mubiru F, Nakalema S, et al. Describing point of entry into care and being lost to program in a cohort of HIV positive pregnant women in a large urban centre in Uganda. *AIDS Research and Treatment.* 2017;2017:3527563.

28. Mwapasa V, Joseph J, Tchereni T, Jousset A, Gunda A. Impact of mother-infant pair clinics and short-text messaging service (SMS) reminders on retention of HIV-infected women and HIV-exposed infants in eMTCT care in Malawi: A cluster randomized trial. *Journal of Acquired Immune Deficiency Syndromes.* 2017;75:S123-S131.

29. Myer L, Phillips TK, Zerbe A, et al. Integration of postpartum healthcare services for HIV-infected women and their infants in South Africa: A randomised controlled trial. *PLoS Medicine.* 2018;15(3):e1002547.

30. Nance N, Pendo P, Masanja J, et al. Short-term effectiveness of a community health worker intervention for HIV-infected pregnant women in Tanzania to improve treatment adherence and retention in care: A cluster-randomized trial. *PloS One.* 2017;12(8):e0181919.

31. Ndatimana D, Ndayisaba G, Bobrow E, et al. Retention-in-care from delivery through 18 months postpartum among HIV-positive mothers receiving lifelong ART in Kigali, Rwanda. International AIDS Society Meeting; 2016; Durban, South Africa.

32. Odeny TA, Hughes JP, Bukusi EA, et al. Text messaging for retention in PMTCT: A stepped-wedge cluster-randomized trial. *Topics in Antiviral Medicine.* 2018;26:365s.

33. Olwedo MA, Lukoda N, Crandall B. Retention of mother-baby pairs in care and treatment through mother-baby care point initiative in Eastern Uganda. *Journal of the International AIDS Society.* 2016;19:232.

34. Oyeledun B, Phillips A, Oronsaye F, et al. The effect of a continuous quality improvement intervention on retention-in-care at 6 months postpartum in a PMTCT program in northern Nigeria: Results of a cluster randomized controlled study. *Journal of Acquired Immune Deficiency Syndromes (1999).* 2017;75 Suppl 2:S156-S164.

35. Phiri S, Tweya H, van Lettow M, et al. Impact of facility- and community-based peer support models on maternal uptake and retention in Malawi's option B+ HIV prevention of mother-to-child transmission program: A 3-arm cluster randomized controlled trial (PURE Malawi). *Journal of Acquired Immune Deficiency Syndromes (1999).* 2017;75 Suppl 2:S140-S148.

36. Sarko KA, Blevins M, Ahonkhai AA, et al. HIV status disclosure, facility-based delivery and postpartum retention of mothers in a prevention clinical trial in rural Nigeria. *International Health.* 2017;9(4):243-251.

37. Schnack A, Rempis E, Decker S, et al. Prevention of mother-to-child transmission of HIV in option B+ era: Uptake and adherence during pregnancy in Western Uganda. *AIDS Patient Care STDS.* Mar 2016;30(3):110-118.

38. Schwartz SR, Clouse K, Yende N, et al. Acceptability and feasibility of a mobile phone-based case management intervention to retain mothers and infants from an option B+ program in postpartum HIV care. *Maternal and Child Health Journal.* 2015;19(9):2029-2037.

39. Tweya H, Gugsa S, Hosseinipour M, et al. Understanding factors, outcomes and reasons for loss to follow-up among women in Option B plus PMTCT programme in Lilongwe, Malawi. *Tropical Medicine & International Health.* 2014;19(11):1360-1366.
